# Hepatoprotective effects of Niudali (*Callerya speciosa*) root aqueous extracts against tetrachloromethane‐induced acute liver injury and inflammation

**DOI:** 10.1002/fsn3.3626

**Published:** 2023-08-17

**Authors:** Yizi Zhang, Jinwen Huang, Lishe Gan, Rihui Wu, Jingwei Jin, Tinghan Wang, Shili Sun, Zhenbiao Zhang, Liya Li, Xi Zheng, Kun Zhang, Lingli Sun, Hang Ma, Dongli Li

**Affiliations:** ^1^ School of Biotechnology and Health Sciences Wuyi University Jiangmen China; ^2^ International Healthcare Innovation Institute (Jiangmen) Jiangmen China; ^3^ Bioactive Botanical Research Laboratory, Biomedical and Pharmaceutical Sciences, College of Pharmacy University of Rhode Island Kingston Rhode Island USA; ^4^ Tea Research Institute, Guangdong Academy of Agricultural Sciences/Guangdong Key Laboratory of Tea Resources Innovation & Utilization Guangzhou China; ^5^ Institute of Microbial Pharmaceuticals, College of Life and Health Sciences Northeastern University Shenyang China

**Keywords:** anti‐inflammation, antioxidant, *Callerya speciosa*, hypaphorine, liver protection, polysaccharides

## Abstract

Niudali (*Callerya speciosa*) is commonly grown in southeastern regions of China and consumed as a food ingredient. Although Niudali root extracts showed various biological activities, the hepatoprotective effects of Niudali root phytochemicals are not fully studied. Herein, we prepared two Niudali root aqueous extracts, namely, c and Niudali polysaccharides‐enriched extract (NPE), and identified an alkaloid, (hypaphorine) in NEW. The hepatoprotective effects of NWE, NPE, and hypaphorine were evaluated in an acute liver injury model induced by carbon tetrachloride (CCl_4_) in mice. Pathohistological examination and blood chemistry assays showed that treatment of NWE, NPE, and hypaphorine alleviated CCl_4_‐induced liver damage by lowering the liver injury score (by 75.51%, 80.01%, and 41.22%) and serum aspartate and alanine transaminases level (by 63.24%, 85.22%, and 49.74% and by 78.73%, 80.08%, and 81.70%), respectively. NWE, NPE, and hypaphorine also reduced CCl_4_‐induced hepatic oxidative stresses in the liver tissue by decreasing the levels of malondialdehyde (by 40.00%, 51.25%, and 28.75%) and reactive oxygen species (by 30.22%, 36.14%, and 33.54%) while increasing the levels of antioxidant enzymes including superoxide dismutase (by 21.36%, 21.64%, and 8.90%), catalase (by 22.13%, 33.33%, and 5.39%), and glutathione (by 84.87%, 90.65%, and 80.53%), respectively. Mechanistic assays showed that NWE, NPE, and hypaphorine alleviated liver damage by mediating inflammatory biomarkers (e.g., pro‐inflammatory cytokines) via the signaling pathways of mitogen‐activated protein kinases and nuclear factor‐κB. Findings from our study extend the understanding of Niudali's hepatoprotective effects, which is useful for its development as a dietary intervention for liver inflammation.

## Graphical abstract

Niudali (*Callerya speciosa*) root aqueous extracts including a water extract (NWE) and a polysaccharides‐enriched extract (NPE) were prepared and standardized. NWE and NPE exerted hepatoprotective effects against tetrachloromethane‐induced acute liver injury.

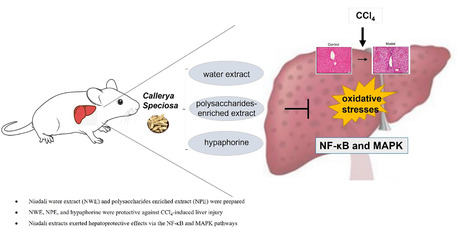

## 1 INTRODUCTION

The liver is a major organ with vital biological functions including the biosynthesis of essential proteins and biochemicals for digestion and growth. It is also the largest organ for the detoxification of exogenous detrimental substances by producing toxin‐metabolizing enzymes to convert harmful xenobiotics into low‐toxic metabolites (Ben Hsouna et al., 2023; Ben Hsouna, Hfaiedh, et al., 2022). Numerous free radicals, such as reactive oxygen species (ROS) and reactive nitrogen species (RNS), are generated during the detoxification process (Forrester et al., 2018; Fransen et al., 2012). Although these free radicals are important molecules involved in cellular defense mechanisms against pathogens, excessive ROS can lead to hepatic cells and tissue damage (Ben Hsouna, Dhibi, et al., 2022). Among the xenobiotics, carbon tetrachloride (CCl_4_), an industrial substance as a dry‐cleaning agent or a fire suppressant, is a potent chemical toxin that can induce severe liver damage by triggering the cytochrome P450 enzymes in the liver to generate a specific type of ROS, namely, trichloromethyl and proxy chloromethyl free radicals (Ben Hsouna, Dhibi, Dhifi, Mnif, et al., 2019; Ben Hsouna, Gargouri, Dhifi, Ben Saad, et al., 2019; Ben Hsouna, Gargouri, Dhifi, & Saibi, 2019; Hsouna et al., 2019). Moreover, CCl_4_‐induced oxidative stress can trigger a series of immune responses including the secretion of pro‐inflammatory cytokines such as interleukins (ILs), tumor necrosis factor‐alpha (TNFα), and nuclear factor kappa B (NF‐κB) (Gupta et al., 2022). Thus, CCl_4_‐induced liver damage is a well‐established experimental model for the biological assessment of acute inflammatory liver diseases.

The hepatoprotective effects against CCl_4_‐induced liver damage by natural products are supported by numerous published studies (Pan et al., 2020; Parthasarathy & Evan Prince, 2021; Ugwu & Suru, 2021). Different classes of dietary natural products may modulate the endogenous antioxidant system to ameliorate toxins‐induced liver damage. For instance, silymarin, a group of flavonolignans (including silibinin) from the medicinal plant milk thistle (*Silybum marianum*), was reported to exert liver protective effects by counteracting alcohol‐induced enzyme activity changes for SOD, glutathiones‐transferase (GST), CAT, glutathione reductase (GR), and GPx in a rodent model (Das & Vasudevan, 2006). In addition, similar hepatoprotective effects were reported from other chemotypes of dietary natural products such as polysaccharides. Treatment with water‐soluble apple peel polysaccharides (250 and 500 mg/kg body weight/per day) reduced CCl_4_‐induced hyperactivity of oxidative enzymes (i.e., serum alanine aminotransferase, aspartate aminotransferase, and lactic dehydrogenase) in a mice model (Yang et al., 2013). Additionally, alkaloids from medicinal plants, such as curry leaves (*Murraya koenigii*) (Sangale & Patil, 2017) and Jerusalem cherry (*Solanum pseudocapsicum*) (Sangale & Patil, 2017), were also reported to confer hepatoprotective effects against CCl_4_‐induced liver injuries in rats. Our previously reported study identified a diverse group of bioactive compounds including polyphenols, alkaloids (such as hypaphorine), terpenoids, and polysaccharides. We also reported the extraction and chemical characterization of crude polysaccharides from a hot water extract of Niudali. Moreover, the Niudali water extract was shown to exert alleviative effects against metabolic disorders and gut microbiota dysbiosis in a mice model (Li et al., 2022). However, bioactive phytochemicals from Niudali with hepatoprotective are not fully elucidated. Herein, we aim to evaluate whether a Niudali water extract (NWE) and Niudali polysaccharides‐enriched extract (NPE), and identified an alkaloid, (hypaphorine) can protect the liver from CCl_4_‐induced acute liver injury in mice. Furthermore, possible mechanisms of action of them were explored by assessing their modulatory effects on a series of molecular targets including enzymes and signaling pathways using in vivo assays.

## 2 MATERIALS AND METHODS

### 2.1 Materials

The roots of *C. speciosa* were purchased from a local market (Jiangmen, Guangdong, China) and air‐dried. The dried root material (100 g) was extracted with distilled water at 100°C twice (with 10 volumes of water for 1 h each time). The aqueous extract of *C. speciosa* roots was filtered, concentrated, and freeze‐dried to afford a Niudali water extract (NWE) with a yield of 17.65%. Apart from NWE, two samples including a polysaccharides‐enriched extract further purified from NWE (Niudali polysaccharides extract; NPE) as we reported (Li et al., 2022) and hypaphorine, an indole alkaloid that was previously isolated from *C. speciosa* roots (Dongli; Li et al., 2021), were included in this study.

### 2.2 Determination of total carbohydrate and hypaphorine content in NWE

The chemical constituents, including the content of total carbohydrate and hypaphorine of NWE, were characterized. First, the total carbohydrate content of NWE was determined by a colorimetric (phenol‐sulfuric acid) method using glucose as a standard curve (Chen & Huang, 2018). Briefly, 20.0 mg of NWE was accurately weighed and dissolved with distilled water to obtain an NWE solution at a concentration of 0.1 mg/mL. This NWE solution (200 μL) was mixed with phenol‐sulfuric acid reagent, and the mixture was kept at room temperature for 20 min. Distilled water was used as a blank control, and the absorbance of the reaction products was measured at a wavelength of 490 nm to determine the carbohydrate content.

The hypaphorine level of NWE was determined by a chromatographic method performed on a Waters UPLC H‐class system coupled with a DAD detector. The separation of NWE was achieved on an ACQUITY‐UPLC‐BEH‐C18 column (100 × 2.1 mm; 1.7 μm) at a flow rate of 0.3 mL/min with a detection wavelength of 218 nm. The gradient elution system consisting of solvent A (water) and solvent B (acetonitrile) was set as follows: 0–3 min, 0%–20% B; 3–4.5 min, 20%–40% B; 4.5–6 min, 40%–60% B; 6–8 min, 60% B; 8.05–10 min, 0% B. The injection volume for each analysis was 1 μL and the temperature of the column was kept at 30°C. All samples were filtered through a membrane filter (0.22 μm).

### 2.3 Establishment of a murine liver injury model

All procedures in the animal experiments were conducted in accordance with the Animal Care and Use Guidelines of Tea Research Institute Guangdong Academy of Agricultural Sciences, and approved by the Institutional Animal Care and Use Committee. Male C57BL/6 mice (aged 7 weeks) were purchased from the Dien Gene Technology Co. Ltd. (Guangzhou, Guangdong, China) and housed at an environment of 60%–70% humidity and room temperature (22 ± 2°C) under a 12 h light/dark cycle. Food and water were provided ad libitum. After 1 week of acclimatization, 8‐week‐old mice (60) were randomly assigned to six groups (*n* = 10 each): (1) control group (normal), (2) model group (exposed to CCl_4_), and treatment groups including (3) CCl_4_ + silymarin (100 mg/kg/body weight; positive control), (4) CCl_4_ + NWE (500 mg/kg/body weight), (5) CCl_4_ + NPE (50 mg/kg/body weight), and (6) CCl_4_ + hypaphorine (20 mg/kg/body weight). On the first day of the experiment, the model and treatment groups were intraperitoneally injected with a CCl_4_ solution (2%) dissolved in maize oil to induce liver injury. On day 2, mice in the treatment groups were administrated samples by gavage once a day for 3 days, and the control and model groups received the same volume of water. Two hours after the last treatment, the body weights of the mice were measured before their euthanization. Blood and liver were collected for further analysis. The wet weight of the liver tissues was weighed, and the liver index of each sample was calculated by the following formula: liver index (%) = liver wet weight/mouse body weight ×100%.

### 2.4 Assessment of liver injury

The severity of the liver injury was scored from one to five according to the degree of necrosis, coagulative central area, and focal. The degree of lesions was assessed from one to five depending on severity: a score of 0 for normality, 1 for minimal (<1%), 2 for slight (1%–25%), 3 for moderate (26%–50%), 4 for moderate/severe (51%–75%), and 5 for severe (76%–100%) (Wu et al., 2019).

### 2.5 Histological staining

The fixed liver tissues were cut into blocks (3 mm^3^) and embedded in paraffin. The tissue blocks were cut into 5 μm thick sections with a paraffin microtome (Leica, Switzerland), which were then deparaffinized twice with xylene (Damao, Tianjin, China) and subsequently rehydrated with 100%, 95%, 80%, and 70% of aqueous ethanol (Damao, Tianjin, China). The consecutive sections were stained with staining agents including hematoxylin and eosin (H&E) (Shanghai Beyotime Biotechnology) and Masson trichromic (Solarbio, Beijing, China) according to the manufacturer's instructions. The stained sections were dehydrated (with 95% and 100% ethanol), washed twice with xylene, sealed with neutral resin, and observed under a light microscope (Olympus, Japan).

### 2.6 Blood biochemical assays

Frozen liver tissues were thawed and homogenized in physiological saline solution on ice using a homogenizer (OMNI Bead Ruptor 24, America) and centrifuged at 2500 rpm and 4°C for 10 min. The protein content in the supernatant was measured using the Pierce BCA protein assay kit (Thermo VK312556, America). The levels of biomarkers including alanine aminotransferase (ALT), aspartate aminotransferase (AST), malondialdehyde (MDA), catalase (CAT), superoxide dismutase (SOD), and glutathione (GSH) (Jiancheng Company; Nanjing, China) were measured using specific assay kits according to the manufacturer's instructions. The reactive oxygen species (ROS) level was measured using an ELISA kit (Meimian; Jiangsu, China).

### 2.7 Immunohistochemistry (IHC) assay

The paraffin sections of tissues were incubated with H_2_O_2_ (3%; Solarbio; Beijing, China) to quench the endogenous peroxidases and then kept at 4°C in ethylene diamine tetraacetic acid (EDTA) (Solarbio) to unmask the antigens. After blocking with goat serum (5%; Solarbio) for 30 min, the sections were incubated with primary antibodies of interleukin‐6 (IL‐6), cyclooxygenase‐2 (COX‐2), tumor necrosis factor‐α (TNF‐α), nitric oxide synthase (iNOS), phospho‐NF‐κB‐p65 (Ser536), nuclear factor kappa‐B (NF‐κB), and inhibitor of NF‐κB (IκB) for overnight at 4°C. The sections were then probed with the respective secondary antibody for 1 h, followed by adding the streptavidin‐biotin complex (SABC) reagent (Beyotime Biotechnology, Shanghai, China) for 30 min. After the staining development with diaminobenzidine (DAB) (Shanghai Beyotime Biotechnology) for 2 to 5 min, the sections were counterstained with hematoxylin (Shanghai Beyotime Biotechnology) for 90 s, and then dehydrated through an ethanol gradient (80%–100%), cleared with xylene, mounted with neutral resin, and observed under a light microscope (Olympus, Japan).

### 2.8 Western blotting assays

The liver tissues were homogenized in the RIPA lysis buffer (Shanghai Beyotime Biotechnology) and kept in an ice bath for 1 h followed by centrifugation at 4°C for 20 min at 13,200 rpm. The protein content in the supernatants was measured using the Pierce BCA protein assay kit (Thermo VK312556). An equal amount of protein per sample was boiled in the SDS‐PAGE loading buffer (4× with DTT) for 5 min, and 20 μL of the protein solution was used for the display of SDS‐PAGE. The protein bands were transferred to the PVDF membranes (Millipore; Burlington, MA, USA), and blocked with skim milk (5%) or BSA in TBST (Tris‐buffered saline containing 0.1% of tween‐20) at room temperature for 2 h. The blots were incubated with primary antibodies against IL‐6, COX‐2, TNF‐α, iNOS, p‐NF‐κB, and IκBα, phospho‐p44/42 MAPK (Erk1/2), p44/42 mitogen‐activated protein kinase (p44/42 MAPK), phospho‐p38 MAPK (Thr180/Tyr182), p38 mitogen‐activated protein kinase (p38 MAPK), Phospho‐SAPK/JNK (Thr183/Tyr185), c‐Jun N‐terminal kinase (JNK), B‐cell lymphoma‐2 (Bcl‐2), BCL2‐associated X protein (Bax), and *β*‐actin separately for overnight at 4°C. After washing with TBST (5 min) three times, the membrane was incubated with the secondary antibody (KPL) for 50 min. The bands were developed in the dark for 2 min and imaged using a chemiluminescence gel imaging system (Tanon 5200; Shanghai, China).

### 2.9 Statistical analysis

All data are presented as the mean ± SEM of at least three independent experiments. The Shapiro‐Wilk normality test was used to assess the distribution of the data. When the data showed a normal distribution, parametric tests were used. The means were compared by one‐way ANOVA followed by Dunnett's test using GraphPad Prism 8.0 for Windows (GraphPad Software Inc.). For data that showed a non‐normal distribution, the Kruskal‐Wallis test was used. When the *p*‐value was less than .05, the difference between groups was considered statistically significant.

## 3 RESULTS

### 3.1 Quantitation of total carbohydrate and hypaphorine contents in NWE

**FIGURE 1:**
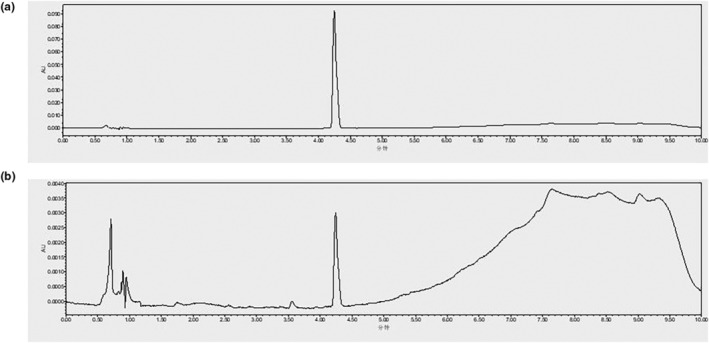
UPLC‐UV profiles of the standard of hypaphorine (a) and a water extract of *C. speciosa* roots (NWE) (b). Detection wavelength: 218 nm; Retention time of hypaphorine was 4.24 min.

A standard curve with glucose as a standard was obtained for the phenol‐sulfuric acid method (with a linearity of *R* ^2^ = 0.9992), and the total carbohydrate content of NWE was determined to be 43.1%. The HPLC‐UV profiles of the hypaphorine standard and NWE are shown in Figure 1. Hypaphorine in NWE was identified by comparison of the retention time of the hypaphorine standard. A calibration curve with a linearity of *R* ^2^ = 0.9999 was obtained, and the level of hypaphorine in NWE was determined as 0.54% (Table 1).

**TABLE 1:** Total carbohydrate and hypaphorine content in water extract of *C. speciosa* roots (NWE).

Sample	Standard curve	*R* ^2^	Content (%)
Total carbohydrate	*y* = 9.8848*x* + 0.0117	0.9992	43.10
Hypaphorine	*y* = 22,932*x* − 939.6	0.9999	0.54

### 3.2 *C. speciosa* extracts ameliorated CCl_4_ ‐induced liver injury

**FIGURE 2:**
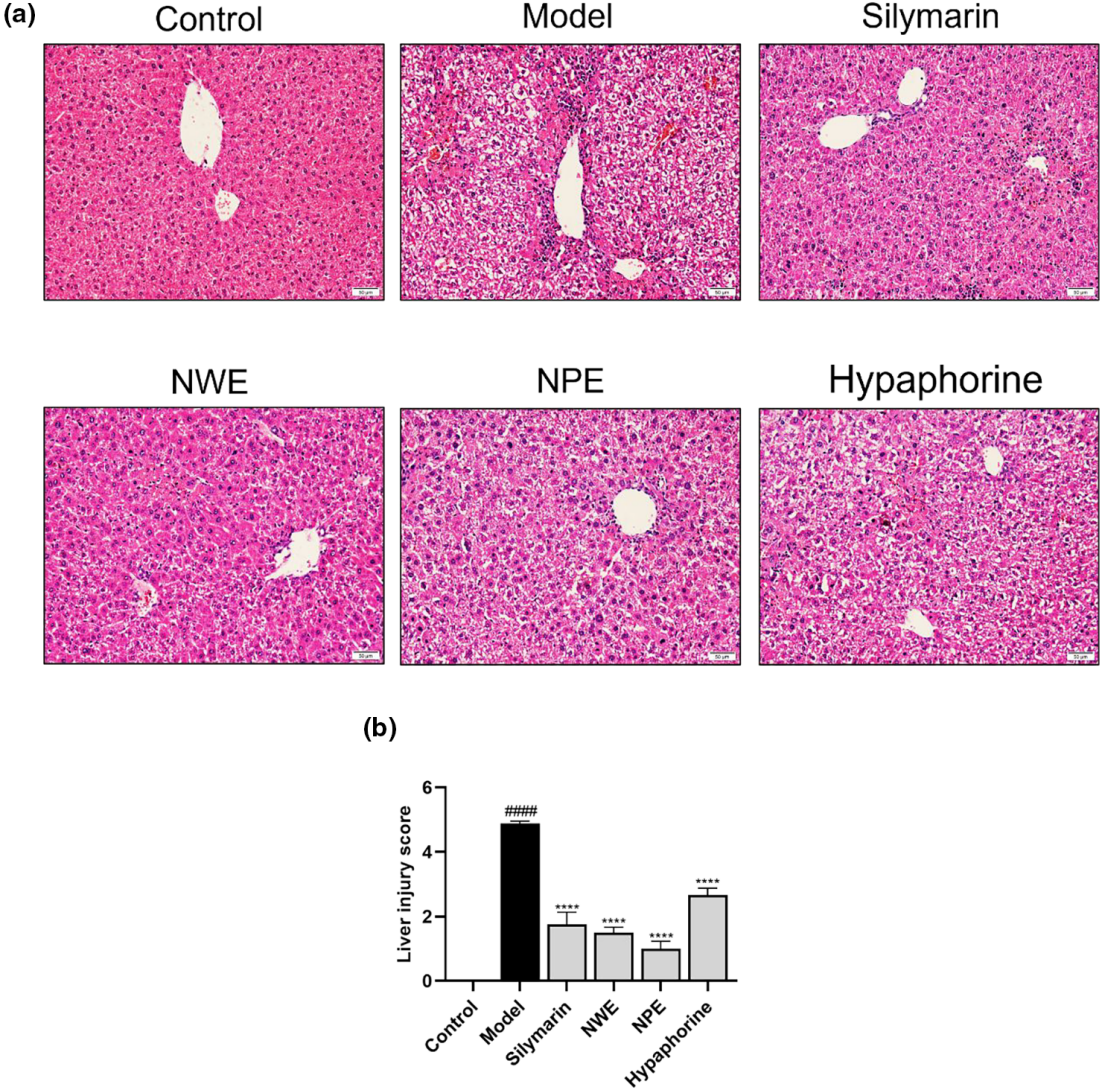
Effects of *C. speciosa* roots on CCl_4_‐induced histopathological examination of liver tissue. The histopathological observation of hematoxylin and eosin (H&E)‐stained liver tissue slices (200×) (a). Liver injury was analyzed by the liver injury scoring system (b). Data are expressed as the mean ± SEM of at least three independent experiments (*n* ≥ 5). ^####^ *p* < .0001 compared to the control group; ****p < .0001 compared to the model group.

Exposure to CCl_4_ induced liver damage in mice, as the liver tissues were enlarged and lusterless as compared to the mouse liver tissues in the control group (See Figure S1). The liver damage was alleviated by the treatments with NWE, NPE, and hypaphorine, as the liver tissue appeared normal. To quantitatively assess NWE’ and NPE's effects on liver functions, histological examinations of the AST and ALT levels in the liver tissues were performed. As shown in Figure 2a, the liver tissue of mice in the control group was intact, as the hepatic lobules were clear and hepatocytes were regularly arranged. The mouse liver function was disrupted by the exposure to CCl_4_, which led to significant liver damage, including massive hepatic necroses, cell swelling, disappearing hepatocyte architecture, and infiltrated inflammation around the central venous lesions. In contrast, treatments with NWE, NPE, and hypaphorine ameliorated CCl_4_‐induced liver injury, as evidenced by the histopathological characteristics. Furthermore, the liver injury score showed that treatments with NWE, NPE, and hypaphorine had liver protective effects against CCl_4_‐induced injury (Figure 2b). The staining of liver fibrosis with Masson trichrome (for visualization of collagen fibers; Figure S2) showed pathological changes, including interstitial fibrosis, in the CCl_4_‐exposed model group. Treatment with NPE exhibited a reduction of cellular degeneration and fibrosis, while the NWE and hypaphorine treatments were inactive. Apart from the histological staining, data from the biochemical assays measuring the levels of ALT and AST supported the hepatoprotective effects. The levels of serum ALT and AST were elevated in the CCl_4_‐exposed group compared to the control group (45.99 vs. 401.41 U/L for ALT and 25.20 vs. 204.82 U/L for AST). Treatment with NWE, NPE, and hypaphorine suppressed CCl_4_‐elevated levels of serum ALT and AST by 63.24%, 85.22%, and 49.74% and by 78.73%, 80.08%, and 81.70%, respectively (Table 2).

**TABLE 2:** Effects of *C. speciosa* root extracts on the levels of ALT and AST in the serum of mice exposed to CCl_4_.

Group	ALT (U/L)	AST (U/L)
Control	45.99 ± 6.83	25.20 ± 4.40
Model	404.41 ± 40.48^####^	204.82 ± 17.43^####^
Silymarin	81.59 ± 9.49^****^	58.16 ± 2.78^****^
NWE	147.50 ± 22.57^****^	43.57 ± 4.41^****^
NPE	59.32 ± 5.35^****^	40.80 ± 2.09^****^
Hypaphorine	201.80 ± 17.99^****^	37.48 ± 4.82^****^

*Note*: Data are expressed as the mean ± SEM of independent experiments (*n* ≥ 5). ^####^ *p* < .0001 as compared to the control group; ****p < .0001 as compared to the model group.

### 3.3 *C. speciosa* extracts alleviated CCl_4_ ‐induced oxidative stress in mice's liver

Insult with CCl_4_ significantly increased the levels of MDA, an indicator of lipid peroxidation, in the liver tissues from 0.26 to 0.80 nmol/mg compared to the control group. Treatment with NWE, NPE, and hypaphorine counteracted CCl_4_‐induced changes by reducing the MDA level to 0.48, 0.39, and 0.57 nmol/mg, respectively (Table 3). A similar trend was observed in an oxidative stress indicator (i.e., ROS) in the liver tissue. Exposure to CCl_4_ led to an increased production of ROS (from 25.89 to 40.40 U/mg), which was ameliorated by the treatment of NWE, NPE, and hypaphorine (30.22%, 36.14%, and 33.54%, respectively; Table 3). Moreover, NWE, NPE, and hypaphorine restored CCl_4_‐decreased levels of antioxidant enzymes including CAT, SOD, and GSH by 22.13%, 33.33%, and 5.39%, by 21.36%, 21.64%, and 8.90%, and by 84.87%, 90.65%, and 80.53%, respectively (Table 4).

**TABLE 3:** Effects of *C. speciosa* extracts on the level of MDA and ROS.

Group	MDA (nmol/mg protein)	ROS (U/mg protein)
Control	0.26 ± 0.02	25.89 ± 2.15
Model	0.80 ± 0.03^####^	40.40 ± 1.87^###^
Silymarin	0.45 ± 0.03^****^	29.28 ± 1.56**
NWE	0.48 ± 0.03^****^	28.19 ± 2.55**
NPE	0.39 ± 0.02^****^	25.80 ± 2.26***
Hypaphorine	0.57 ± 0.03^****^	26.85 ± 2.40***

*Note*: Data are expressed as mean ± SEM of independent experiments (*n* ≥ 5). ^###^ *p* < .001 and ^####^ *p* < .0001 as compared to the control group; **p < .01, ***p < .001, and ****p < .0001 as compared to the model group.

**TABLE 4:** Effects of *C. speciosa* root extracts on the level of antioxidants CAT, SOD, and GSH in the liver tissues of CCl_4_‐exposed mice.

Group	CAT (U/mg protein)	SOD (U/mg protein)	GSH (μmol/g protein)
Control	21.71 ± 0.44	110.10 ± 4.45	41.14 ± 2.30
Model	14.10 ± 0.10^####^	78.31 ± 3.43^####^	26.96 ± 1.23^####^
Silymarin	21.18 ± 0.34^****^	84.13 ± 1.77	43.60 ± 2.26^****^
NWE	17.22 ± 0.77^****^	95.04 ± 4.18*	49.84 ± 1.42^****^
NPE	18.80 ± 0.48^****^	95.26 ± 1.79*	51.41 ± 1.81^****^
Hypaphorine	14.86 ± 0.39	85.28 ± 3.02	48.67 ± 1.41^****^

*Note*: Data are expressed as the mean ± SEM of independent experiments (*n* ≥ 5). ^####^ *p* < .0001 as compared to the control group; *p < .05 and ****p < .0001 as compared to the model group.

#### 3.3.1 Speciosa extracts regulate the inflammatory and apoptotic pathways

**FIGURE 3:**
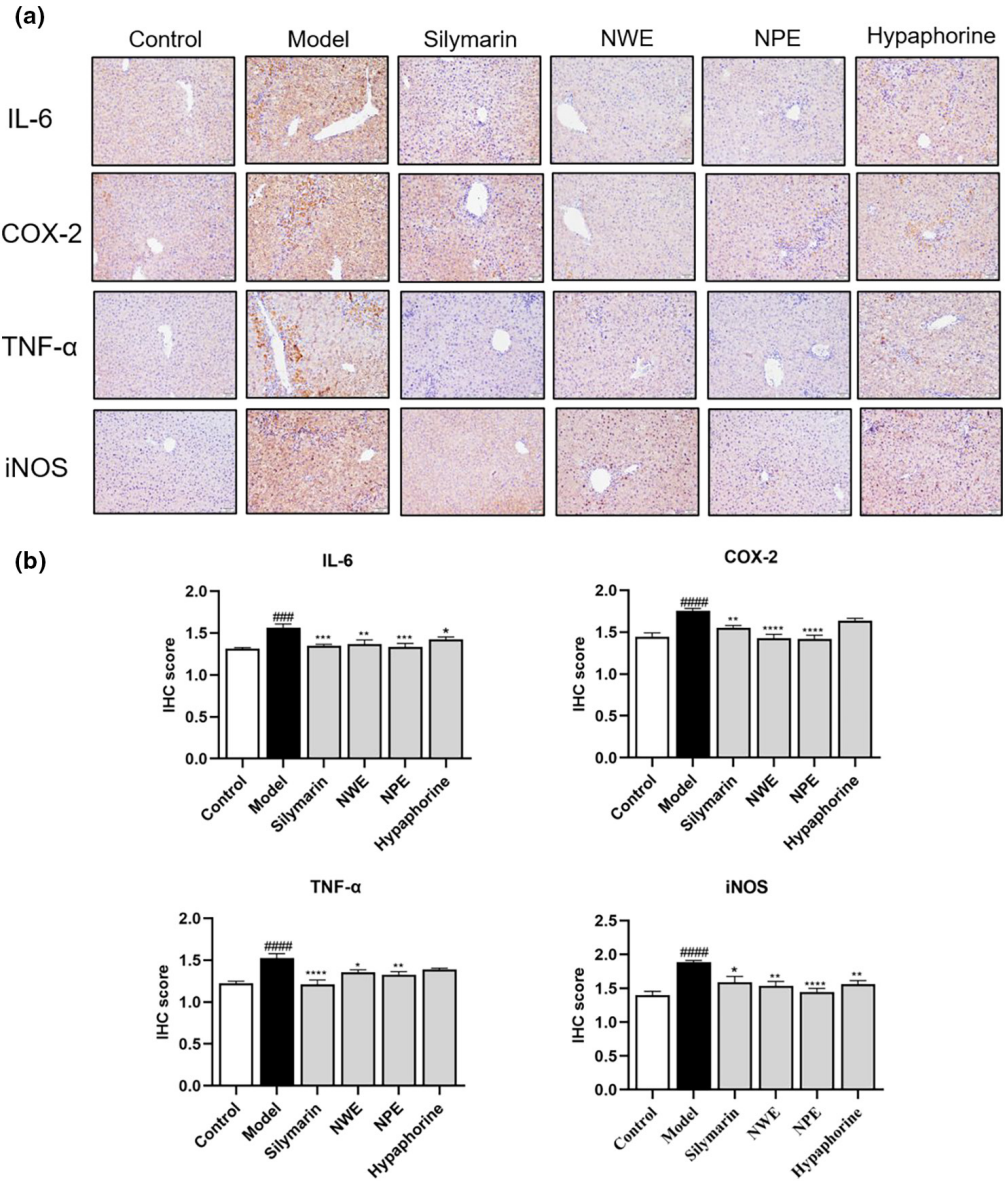
Immunohistochemical staining with IL‐6, COX‐2, TNF‐α, and iNOS in the liver tissues of CCl_4_ treated mice. Representative IHC images of tissue sections showing in situ expression of IL‐6, COX‐2, TNF‐α, and iNOS (a). Quantification of IL‐6, COX‐2, TNF‐α, and iNOS levels (b). Data are expressed as the mean ± SEM of at least three independent experiments (*n* ≥ 5). ^###^ *p* < .001 and ^####^ *p* < .0001 compared to the control group; *p < .05, **p < .01, ***p < .001, and ****p < .0001 compared to the model group.

**FIGURE 4:**
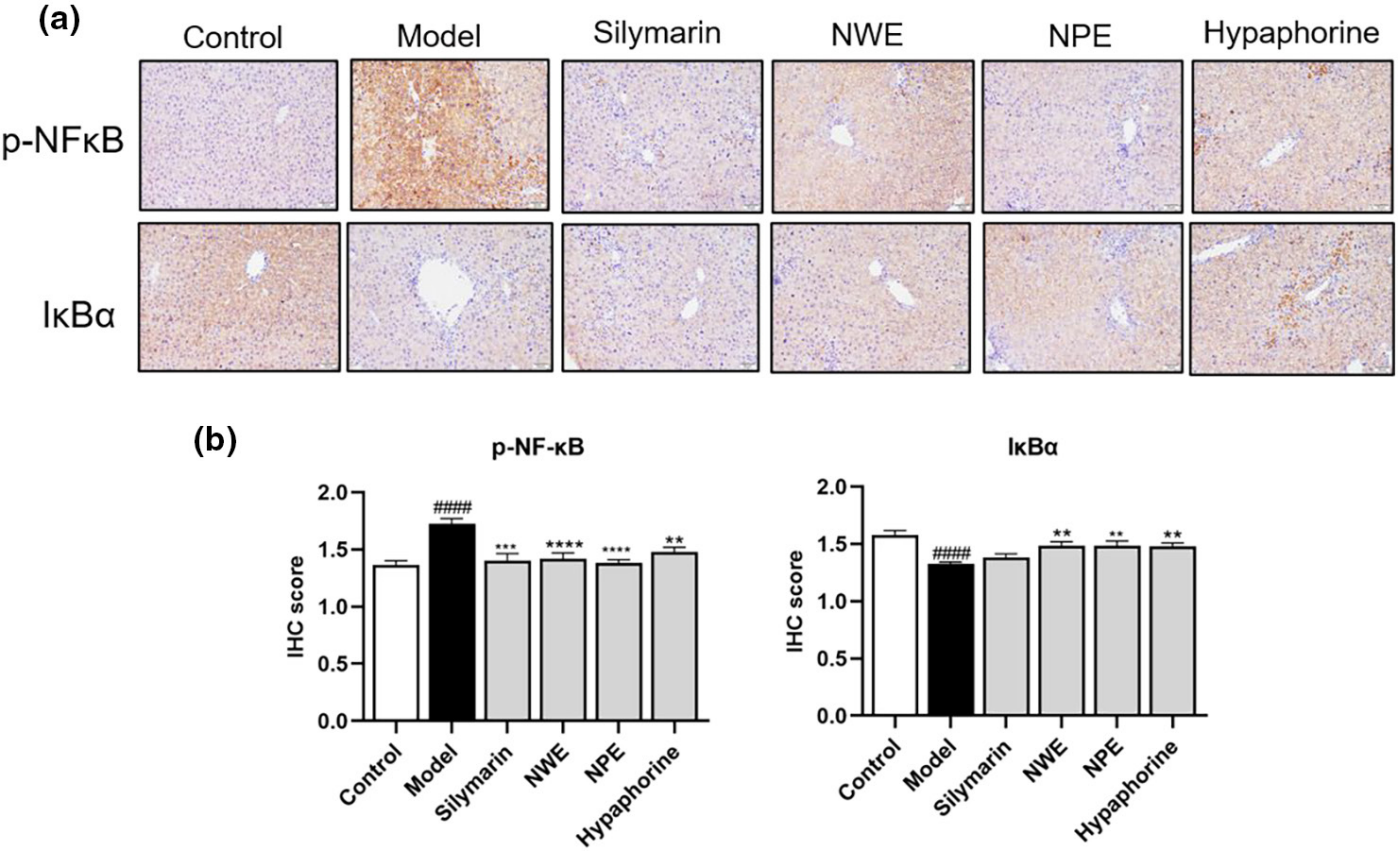
Immunohistochemical staining with p‐NF‐κB and IκBα in the liver tissues of CCl_4_ treated mice. Representative IHC images of tissue sections showing in situ expression of p‐NF‐κB and IκBα (a). Quantification of p‐NF‐κB and IκBα levels (b). Data are expressed as mean ± SEM of at least three independent experiments (*n* ≥ 5). ^###^ *p* < .001 and ^####^ *p* < .0001 compared to the control group; *p < .05, **p < .01, ***p < .001, and ****p < .0001 compared to the model group.

To explore whether *C. speciosa* extracts alleviated inflammation by inhibiting the NF‐κB pathway, the protein levels of inflammatory biomarkers including IL‐6, COX‐2, TNF‐α, iNOS, p‐NF‐κB, and IκBα were determined by immunohistochemistry and western blotting. As shown in Figures 3 and 4, intraperitoneal injection of CCl_4_ significantly increased the expression levels of IL‐6, COX‐2, TNF‐α, iNOS, p‐NF‐κB and decrease IκBα in the inflamed regions of the liver. However, treatment with *C. speciosa* extracts inhibited the CCl_4_‐induced upregulation of IL‐6, COX‐2, TNF‐α, iNOS, p‐NF‐κB and downregulation of IκBα at different degrees compared with the model group.

**FIGURE 5:**
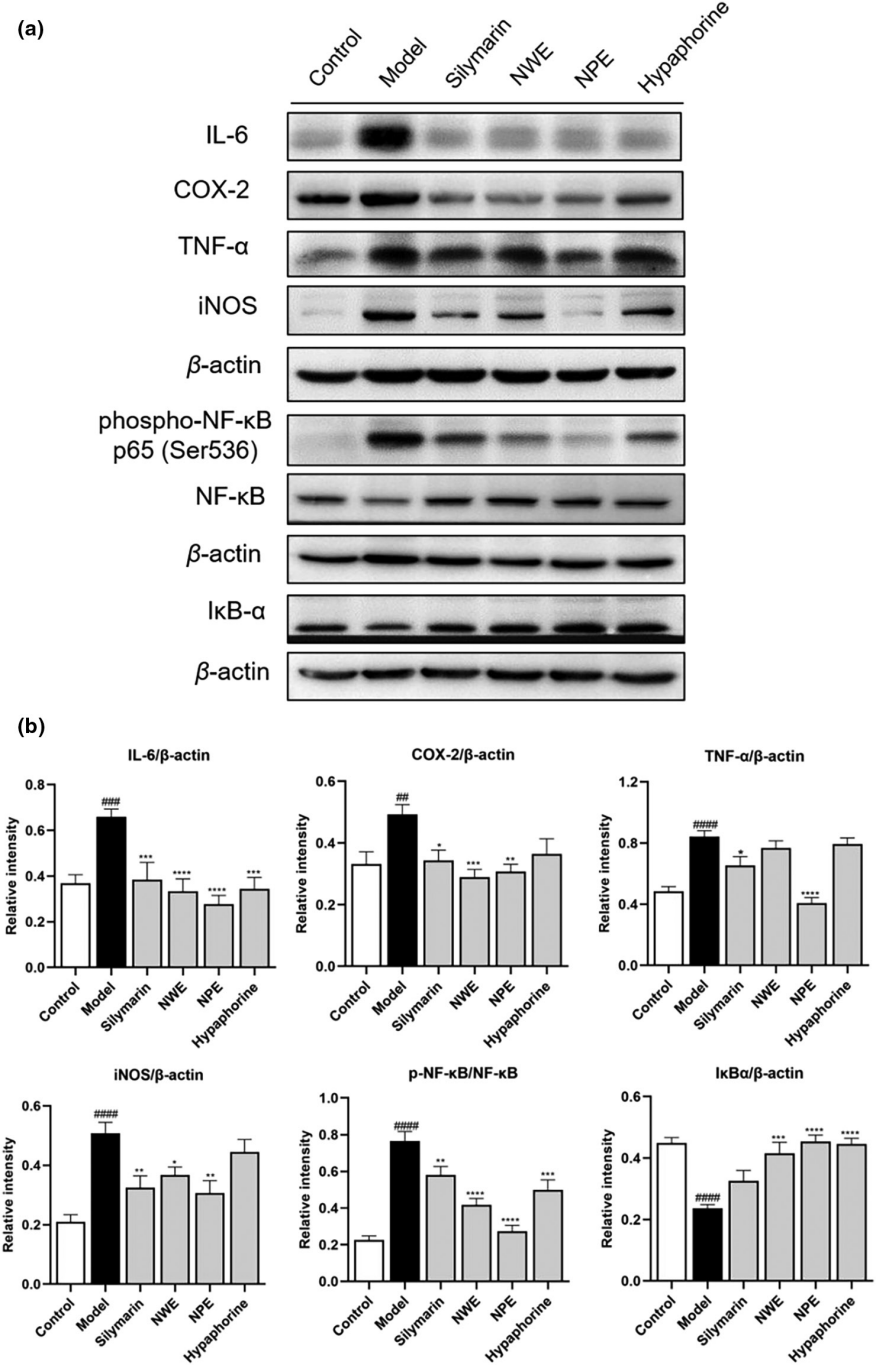
The effect of *C. speciosa* roots on the CCl_4_‐induced inflammatory response in mice. Immunoblot showing expression levels of IL‐6, COX‐2, TNF‐α, iNOS, p‐NF‐κB, NF‐κB, IκBα, and β‐Actin (a). IL‐6, COX‐2, TNF‐α, iNOS, and IκBα normalized to β‐Actin, p65 NF‐κB normalized to NF‐κB (b). Data are expressed as the mean ± SEM of at least three independent experiments (*n* ≥ 3). ^##^ *p* < .01, ###*p* < .001 and ^####^ *p* < .0001 compared to the control group; *p < .05, **p < .01, ***p < .001 and ****p < .0001 compared to the model group.

Insult with CCl_4_ significantly increased the expression levels (relative intensity) of IL‐6, COX‐2, TNF‐α, and iNOS in the inflamed regions of the liver tissue from 0.37 to 0.66, 0.33 to 0.49, 0.48 to 0.84, and 0.21 to 0.51, respectively (Figure 5). The protein expression of p‐NF‐κB increased from 0.23 to 0.77 while the IκBα level decreased from 0.45 to 0.24, respectively by the stimulation of CCl_4_ in the western blot assay (Figure 5). These inflammatory markers were downregulated by the treatment of NWE, NPE, and hypaphorine by 50.00%, 57.58%, and 48.48% for IL‐6, by 40.82%, 36.73%, and 26.53% for COX‐2, by 8.33%, 51.19%, and 6.00% for TNF‐α, by 27.45%, 39.21%, and 13.72% for iNOS, respectively. Treatment with NWE, NPE, and hypaphorine downregulated the expression of p‐NF‐κB by 45.45%, 64.94%, and 35.06%, while upregulating the expression of IκBα by 75.00%, 87.50%, and 87.50%, respectively, as compared to the CCl_4_‐insulted group (Figure 5).

**FIGURE 6:**
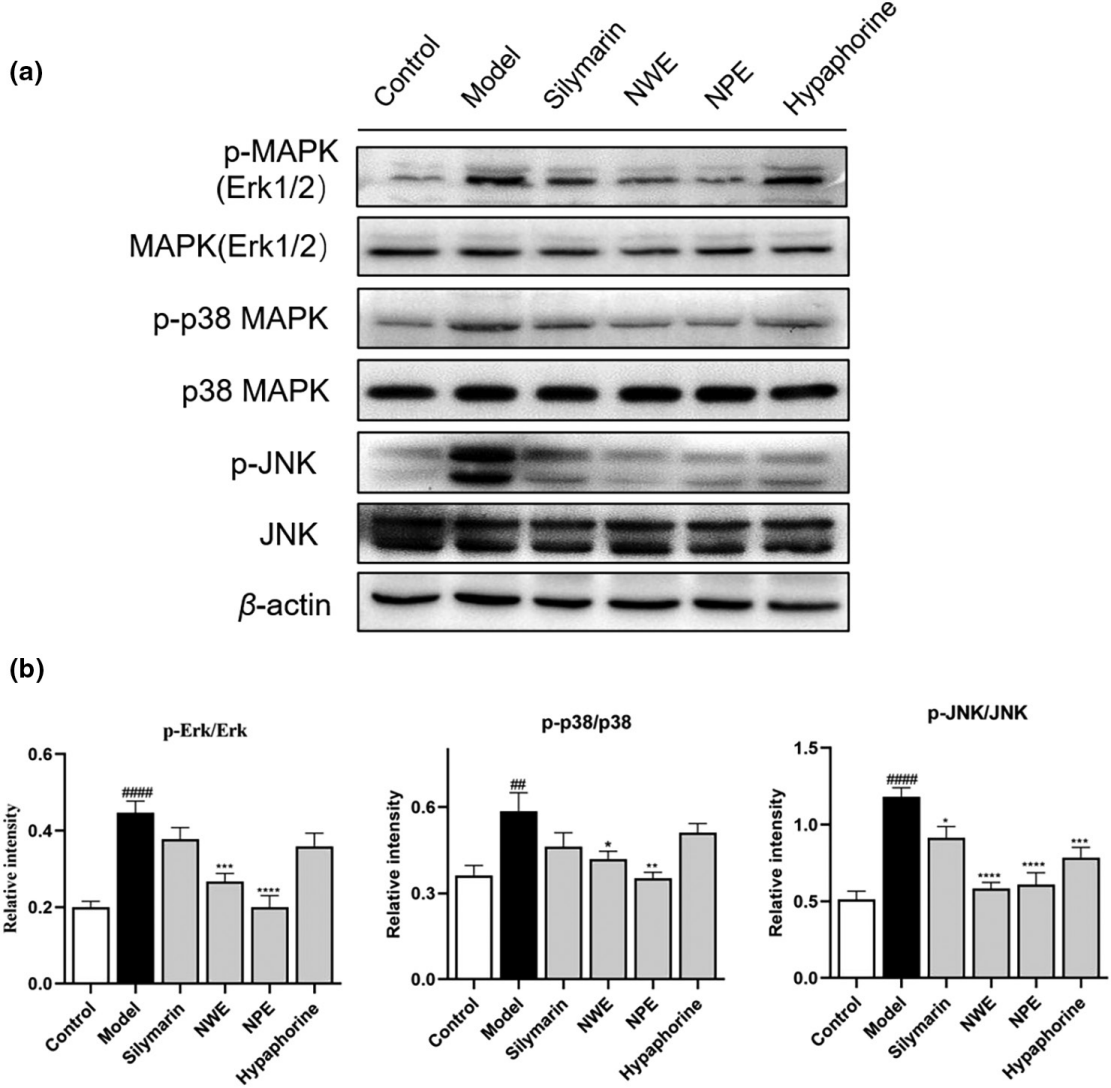
Effect of *C. speciosa* roots on the CCl_4_‐induced inflammatory response in mice. Immunoblot showing expression levels of p‐Erk, Erk, p‐p38, p38, p‐JNK, JNK, and β‐Actin (a). p‐Erk normalized to Erk, p‐p38 normalized to p‐38, p‐JNK normalized to JNK (b). Data are expressed as the mean ± SEM of at least three independent experiments (*n* ≥ 3). ^##^ *p* < .01 and ^####^ *p* < .0001 compared to the control group; *p < .05, **p < .01, ***p < .001, and ****p < .0001 compared to the model group.

Mitogen‐activated protein kinases (MAPKs) signaling pathways play a critical role in the regulation of inflammatory responses and in coordinating the induction of many gene‐encoding inflammatory biomarkers (Zhang & Liu, 2002). The MAPK pathways are mediated by several protein kinases, including ERK1/2, p38, and JNK. Thus, western blot assays were used to evaluate *C. speciosa* extracts' effects on interfering with the MAPK pathways. The effects of *C. speciosa* extracts on phosphorylation of ERK1/2, p38, and JNK were also analyzed. Phosphorylation of ERK1/2, p38, and JNK were elevated in the CCl_4_‐exposed group as their relative intensities in the western blot assay changed from 0.20 to 0.45, 0.36 to 0.56, and 0.51 to 1.18, respectively (Figure 6). Treatment of NWE and NPE reduced the phosphorylation levels of ERK1/2, p38, and JNK by 40.00% and 55.56%, 25.00%, and 37.50%, and 50.85% and 48.31%, while hypaphorine treatment inhibited the phosphorylation of JNK by 33.05% (Figure 6).

**FIGURE 7:**
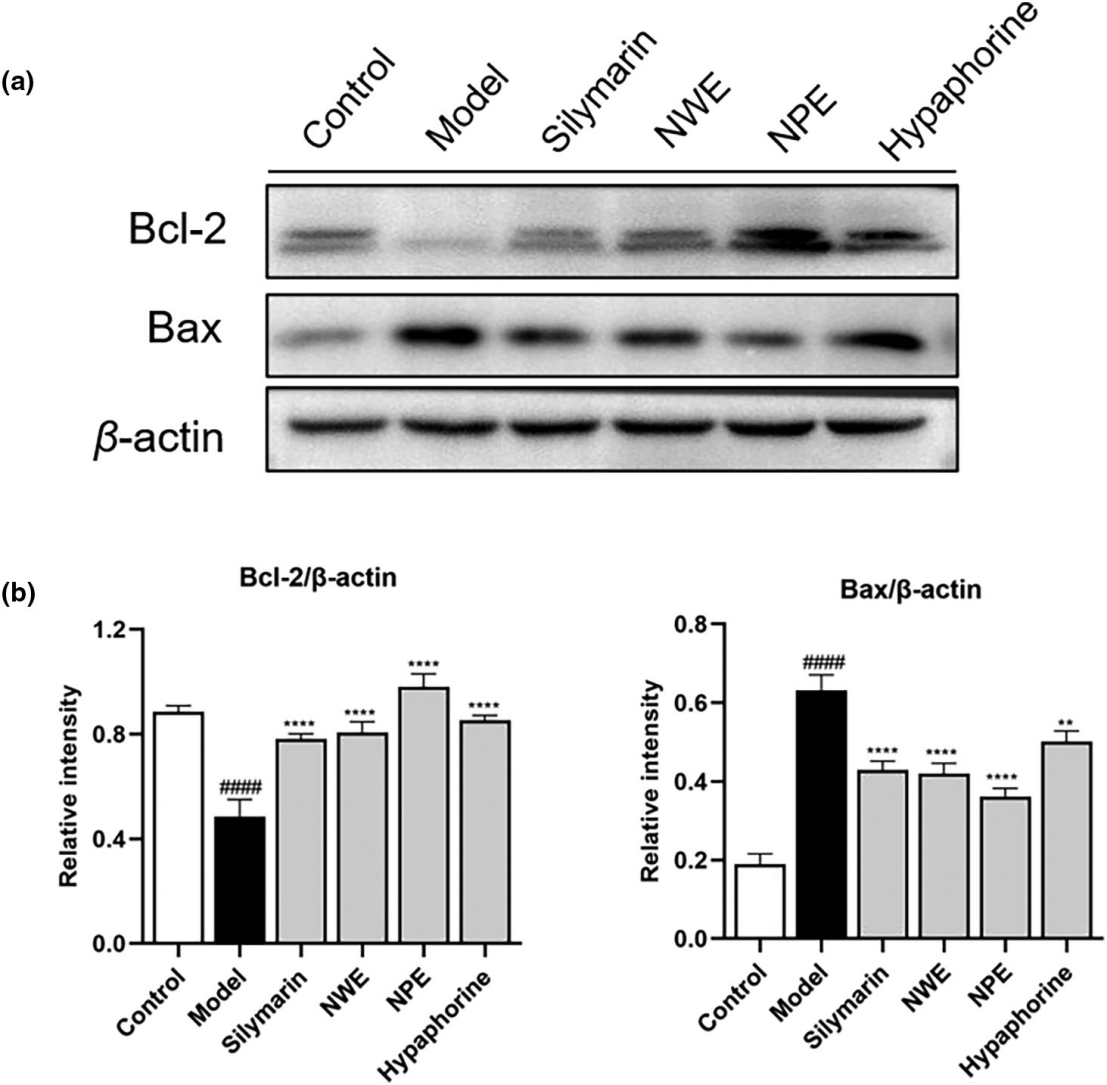
Effect of *C. speciosa* roots on the expression of apoptosis genes in the liver tissues of CCl_4_‐induced mice. Immunoblot showing expression levels of Bax, Bcl‐2, and β‐Actin (a). Bax, Bcl‐2 normalized to β‐Actin (b). Data are expressed as the mean ± SEM of at least three independent experiments (*n* ≥ 3). ^####^ *p* < .0001 compared to the control group; *p < .05, **p < .01****p < .0001 compared to the model group.

Furthermore, treatment of *C. speciosa* extracts also alleviated CCl_4_‐triggered apoptosis in liver cells. Exposure to CCl_4_ resulted in upregulated expression of a pro‐apoptotic protein (i.e., Bax) and downregulated an anti‐apoptotic protein (i.e., Bcl‐2) from 0.19 to 0.63 and from 0.89 to 0.48, respectively (relative intensity) (Figure 7). Treatment with NWE, NPE, and hypaphorine counteracted the apoptotic changes by inhibiting the expression of Bax by 33.33%, 42.86%, and 20.63%, and promoting the level of Bcl‐2 by 68.75%, 106.25%, and 77.08% (Figure 7).

## 4 DISCUSSION

Reported studies showed that Niudali extracts can confer liver protective effects against CCl_4_‐induced inflammation in animal models. For instance, a hot water extract of Niudali showed hepatoprotective effects by ameliorating CCl_4_‐elevated serum ALT and AST levels and reducing the MDA content in mouse liver tissues (Zhou et al., 2009). However, the active compounds in the Niudali water extract that may have contributed to the liver‐protective effects were not identified. Although extensive phytochemical investigation revealed that phenolic compounds are one of the major constituents of Niudali (Zhang et al., 2022), several different classes of phytochemicals, such as alkaloids and polysaccharides, are commonly found in Niudali (Zhang et al., 2021). It is possible that these chemotypes of compounds also contribute to the overall biological effects of Niudali. Indeed, apart from phenolics (including phenolic acids and flavonoids), triterpene saponins (e.g., oleanane‐type triterpenoids) (Wang et al., 2021), alkaloids (e.g., indole, cularine, haplopine, and sanguinarine), and polysaccharides (consisting of monosaccharides including glucose, arabinose, galactose, rhamnose, and galacturonic acid) (Li et al., 2022) have been identified from Niudali extracts. Notably, the triterpene saponin‐enriched Niudali extract showed anti‐inflammatory activity by reducing the levels of pro‐inflammatory cytokines, including IL‐1β and IL‐6 in the liver tissue of high‐fat diet (HFD)‐stimulated mice (Wang et al., 2021). Additionally, polysaccharides from Niudali showed hepatoprotective effects by ameliorating HFD‐induced liver steatosis and inflammation (Li et al., 2022). However, whether polysaccharides and alkaloids from Niudali extract can exert hepatoprotective effects on CCl_4_‐induced liver damage in a mouse model was not well characterized. Therefore, to better understand the hepatoprotective effects of Niudali's phytochemical constituents, we conducted this study to (1) prepare a polysaccharides‐enriched Niudali extract (i.e., NPE), (2) identify an alkaloid, namely, hypaphorine, in NWE compared to the hepatoprotective effects of NWE, NPE, and hypaphorine in a CCl_4_‐induced liver damage model.

Acute liver injury induced by CCl_4_ is associated with the activation of inflammatory processes, oxidative stress, and apoptosis (Al‐Sayed et al., 2019; Sun et al., 2022). In general, treatment with NWE, NPE, and hypaphorine all showed liver protective effects, as evidenced by data from the histopathological evaluation (liver injury score) and the blood chemical assays (ALT and AST level) (Table 2). Reactive oxygen species (ROS) play an important role in regulating physiological and pathophysiological signals. Excessive ROS can lead to oxidative damage, lipid peroxidation, cell membrane damage, degradation, and DNA damage in liver cells (Schraufstatter et al., 1988). ROS activates the NF‐κB response to inflammatory agonists and encodes pro‐inflammatory cytokines by degrading iκB‐α and activating phosphorylation of NF‐κB p65/p50 (Ramadan et al., 2023). The MAPK signaling pathway is also known to be critical for the expression of proinflammatory mediators. Excessive ROS induced by CCl_4_ can activate the MAPK signaling pathways (Liu et al., 2012). Therefore, inhibition of MAPK activation is a possible strategy for the prevention and treatment of liver injury. Compared with the model group, the expression of NF‐κB/MAPK signaling pathway‐related proteins was significantly reduced in the NWE and NPE groups. These results suggest that water extracts of *C. speciosa* roots may regulate the MAPK/NF‐κB pathway to alleviate liver injury. This is supported by studies showing that enzymes including SOD, CAT, and GSH are able to detoxify cellular peroxides (Zhang et al., 2023). The elevated level of a metabolite from lipid peroxidation, namely, MDA, is an indicator of liver damage induced by oxidative stress (Seki et al., 2002). This stress can also be ameliorated by several antioxidative defensive enzymes, including CAT, SOD, and GSH by scavenging lipid peroxides and oxygen‐free radicals (Glade & Meguid, 2017). Treatment with NWE, NPE, and hypaphorine showed liver protective effects in the antioxidant assays measuring the MDA, ROS, and GSH levels (Tables 3 and 4). This suggests that Niudali extracts may protect the liver from CCl_4_‐induced damage by exerting antioxidant effects. Therefore, NWE, NPE, and hypaphorine all showed antioxidant effects against CCl_4_‐induced liver damage, with NPE being the most active Niudali extract, followed by NWE and hypaphorine. Notably, hepatocytes from damaged liver tissue are susceptible to various forms of cell death, including apoptosis and autophagy (Malhi & Gores, 2008). Restricted hepatocyte proliferation may result in liver cirrhosis and an elevated risk of hepatocellular carcinoma (Elkhamesy et al., 2022). Given that CCl_4_ upregulated the pro‐apoptotic factor Bax and decreased the levels of the anti‐apoptotic protein Bcl‐2, which was counteracted by the treatment with NWE, NPE, and hypaphorine, it is possible that *C. speciosa* root extracts may alleviate liver injury via the mediation of apoptosis.

**FIGURE 8:**
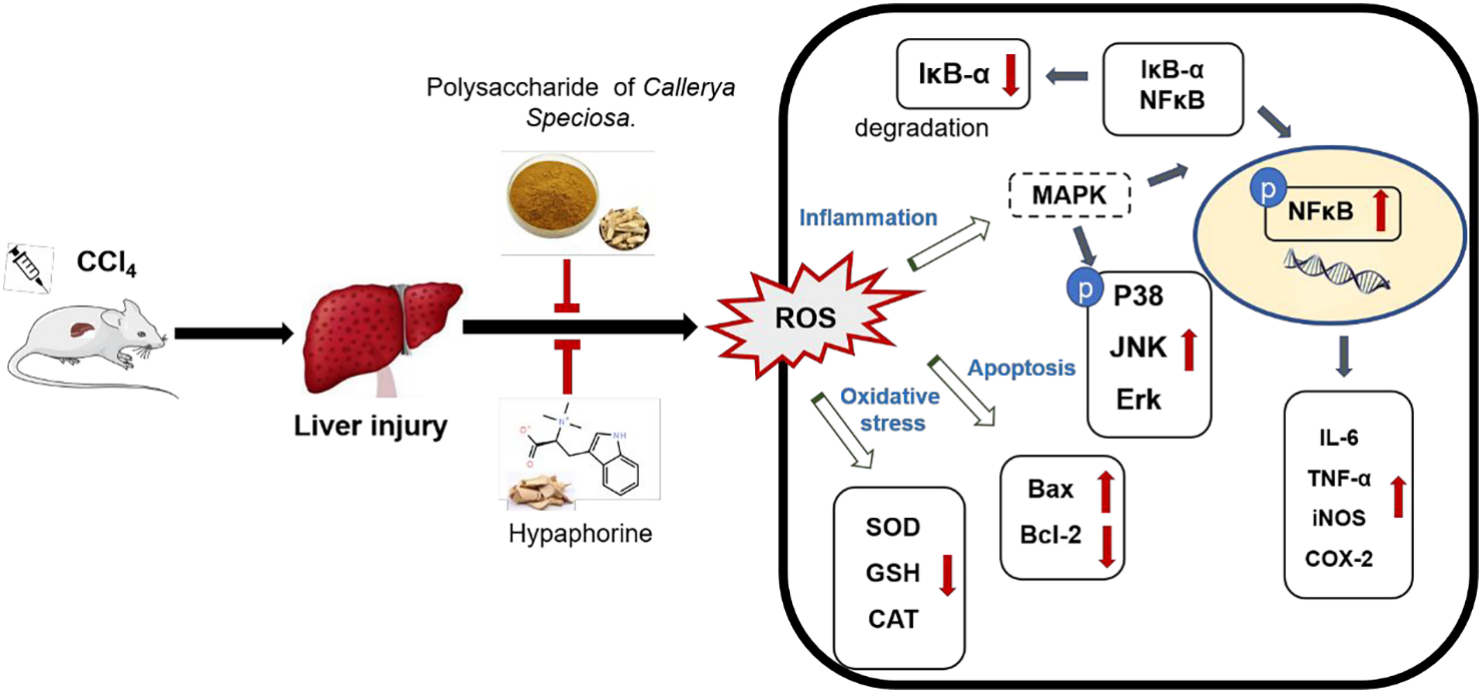
Diagram of proposed hepatoprotective effects of *C. speciosa* roots on CCl_4_‐induced liver injury in mice.

It was noted that hypaphorine was not active in the antioxidant assays measuring the levels of CAT and SOD (Table 4), which suggests that hypaphorine may have distinct mechanisms of action. This was supported by the observation that hypaphorine also showed anti‐inflammatory effects in reducing pro‐inflammatory biomarkers, including IL‐6 (Figures 3b and 5b) and p‐NF‐κB and IκBα (Figures 4 and 5). This was in agreement with reported studies showing that hypaphorine is an anti‐inflammatory compound against lipopolysaccharide‐induced inflammation in the mouse macrophage (RAW 264.7 cells) (Sun, Cai, et al., 2017) and human microvascular endothelia (HMEC‐1 cells) (Sun, Zhu, et al., 2017). Although hypaphorine was not as active as NPE and NWE in several anti‐inflammatory assays (e.g., COX‐2 and TNFα), to date, this is the first in vivo study showing that hypaphorine can alleviate CCl_4_‐induced liver damage. This expands our understanding of the chemotypes of bioactive compounds in Niudali extracts for their hepatoprotective effects. It is noted that, apart from hypaphorine, NPE and NWE both showed promising antioxidant and anti‐inflammatory effects. In particular, the Masson trichrome staining assay showed that CCl_4_‐induced liver fibrosis was ameliorated by the treatment of NPE (Figure S2). This is critical as severe liver fibrosis can lead to cirrhosis, which is irreversible liver damage and a major cause of liver failure and hepatocellular carcinoma (Albanis & Friedman, 2001). Thus, NPE could be a promising dietary intervention for the management of liver diseases, including hepatic fibrosis (Carloni et al., 2014; Cheng et al., 2019; David & Friedman, 2012). This is supported by its effects on the regulation of MAPKs, including ERK1/2, p38, and JNK. These biomarkers are critical for regulating various cellular functions such as cell death, apoptosis, proliferation, and inflammation (Nakagawa & Maeda, 2012). The MAPK signaling pathway also mediates the expression of a series of pro‐inflammatory mediators (Liu et al., 2012). Therefore, inhibition of MAPK activation is a plausible approach to the prevention and treatment of liver injury. Thus, it is possible that Niudali extracts, including NPE and NWE, alleviate liver injury by regulating the MAPK/NF‐κB pathway (Figure 8). However, other mechanisms may also contribute to the overall hepatoprotective effects of polysaccharide‐enriched Niudali extracts. For instance, the gut microbiota‐liver axis may play a pivotal role in Niudali's intervention in liver damage, given that those polysaccharides from Niudali are reported to modulate the gut microbiota and alleviate metabolic disorders in diet‐induced obese C57BL/6 mice (Li et al., 2022). Thus, further studies are warranted to evaluate whether Niudali extracts can alleviate liver injury via the modulation of the gut microbiota.

## 5 CONCLUSION

In summary, we prepared two aqueous Niudali root extracts (i.e., NWE and NPE) and identified an alkaloid (i.e., hypaphorine), as well as evaluated their hepatoprotective effects in a mouse model. We showed that NWE, NPE, and hypaphorine mitigated CCl_4_‐induced liver damage, as evidenced by data from the histopathological and blood chemical assays. Additionally, mechanistic studies on the inflammatory biomarkers suggested that Niudali extracts' liver protective effects were, at least partially, attributed to their modulations of inflammation and apoptosis‐related signaling pathways, including the MAPK/NF‐κB pathway. Notably, both polysaccharides and hypaphorine are active phytochemical constituents contributing to the overall hepatoprotective effects of Niudali extracts. Findings from the study provide useful insights into the bioactive constituents of Niudali extracts and their possible mechanisms of action, which are critical for the further development of Niudali extracts as a dietary intervention for managing liver diseases.

## AUTHOR CONTRIBUTIONS

**Yizi Zhang:** Data curation (lead); formal analysis (lead); writing – original draft (lead). **Jinwen Huang:** Data curation (supporting); formal analysis (supporting); writing – original draft (supporting). **Lishe Gan:** Methodology (equal); supervision (equal). **Rihui Wu:** Methodology (equal); supervision (equal). **Jingwei Jin:** Supervision (equal). **Tinghan Wang:** Writing – review and editing (equal). **Zhenbiao Zhang:** Methodology (lead); supervision (equal). **Liya Li:** Formal analysis (equal); writing – review and editing (supporting). **Lingli Sun:** Funding acquisition (equal); supervision (equal); writing – review and editing (equal). **Hang Ma:** supervision (equal); writing – review and editing (equal). **Dongli Li:** Funding acquisition (equal); supervision (equal); writing – review and editing (equal).

## CONFLICT OF INTEREST STATEMENT

The authors declare that they have no competing interests.

## ETHICS STATEMENT

None (no human subjects in this study).

## Supporting information


Figure S1.
Figure S2.

## Data Availability

The data that support the findings of this study are available on request from the corresponding author.
