# Supplementary material for: Hepatoprotective effects of Niudali (Callerya speciosa) root aqueous extracts against tetrachloromethane‐induced acute liver injury and inflammation
Source: Food Sci Nutr. 2023 Aug 17;11(11):7026–38. doi: 10.1002/fsn3.3626 (PMC10630805; doi:10.1002/fsn3.3626)

**SUPPLEMENTARY MATERIALS**

**Hepatoprotective Effects of** **Niudali (*Callerya Speciosa*) Root Aqueous Extracts against Tetrachloromethane-Induced Acute Liver Injury and Inflammation**

Yizi Zhang ^1^, Jinwen Huang ^1^, Lishe Gan ^1,2^, Rihui Wu ^1,2^, Jingwei Jin ^1,2^, Tinghan Wang ^3^, Shili Sun ^4^, Zhenbiao Zhang ^4^, Liya Li ^5^, Xi Zheng ^1^, Kun Zhang ^1^, Lingli Sun ^4^*, Hang Ma ^1,2,3^*, Dongli Li ^1,2^*

^1^School of Biotechnology and Health Sciences, Wuyi University, Jiangmen 529020, China

^2^International Healthcare Innovation Institute (Jiangmen), Jiangmen 529040, China

^3^Bioactive Botanical Research Laboratory, Biomedical and Pharmaceutical Sciences, College of Pharmacy, University of Rhode Island, Kingston, RI 02881, USA

^4^Tea Research Institute, Guangdong Academy of Agricultural Sciences/Guangdong Key Laboratory of Tea Resources Innovation & Utilization, Guangzhou 510640, China

^5^Institute of Microbial Pharmaceuticals, College of Life and Health Sciences, Northeastern University, Shenyang 110819, China

**Figure S1**. Macroscopic characteristics of livers after treatments of Niudali extracts on CCl4-induced murine model.

**
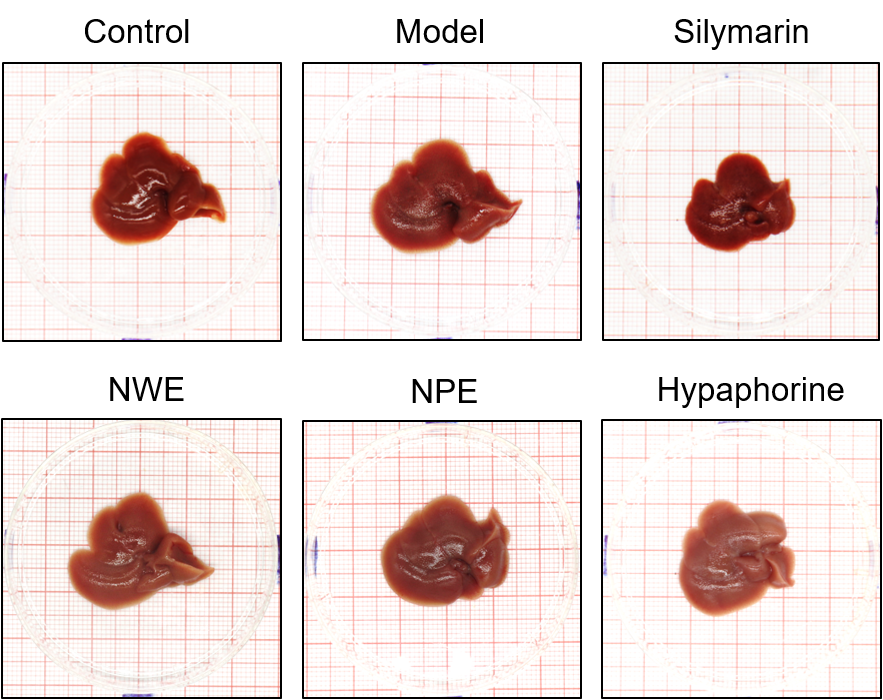
**

**Figure S2**. Representative microscopic photographs of liver sections stained with Masson trichromic.


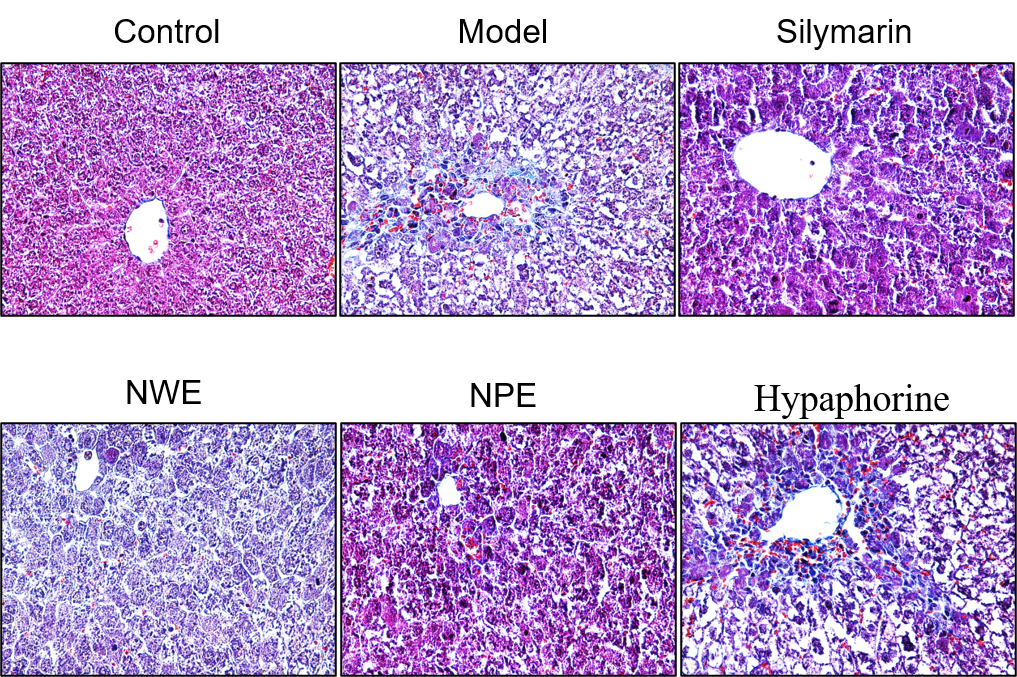


Original Western blot images


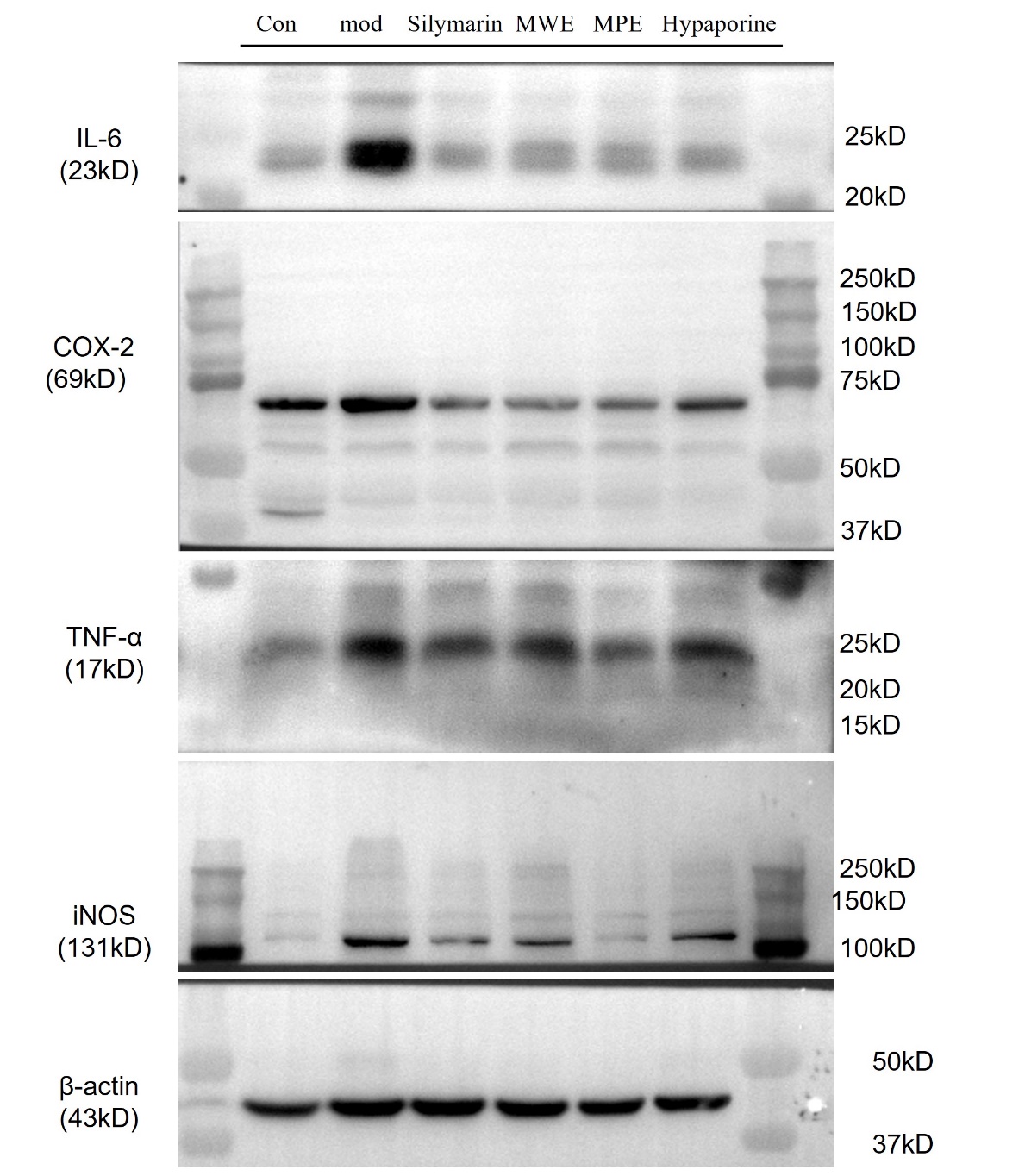


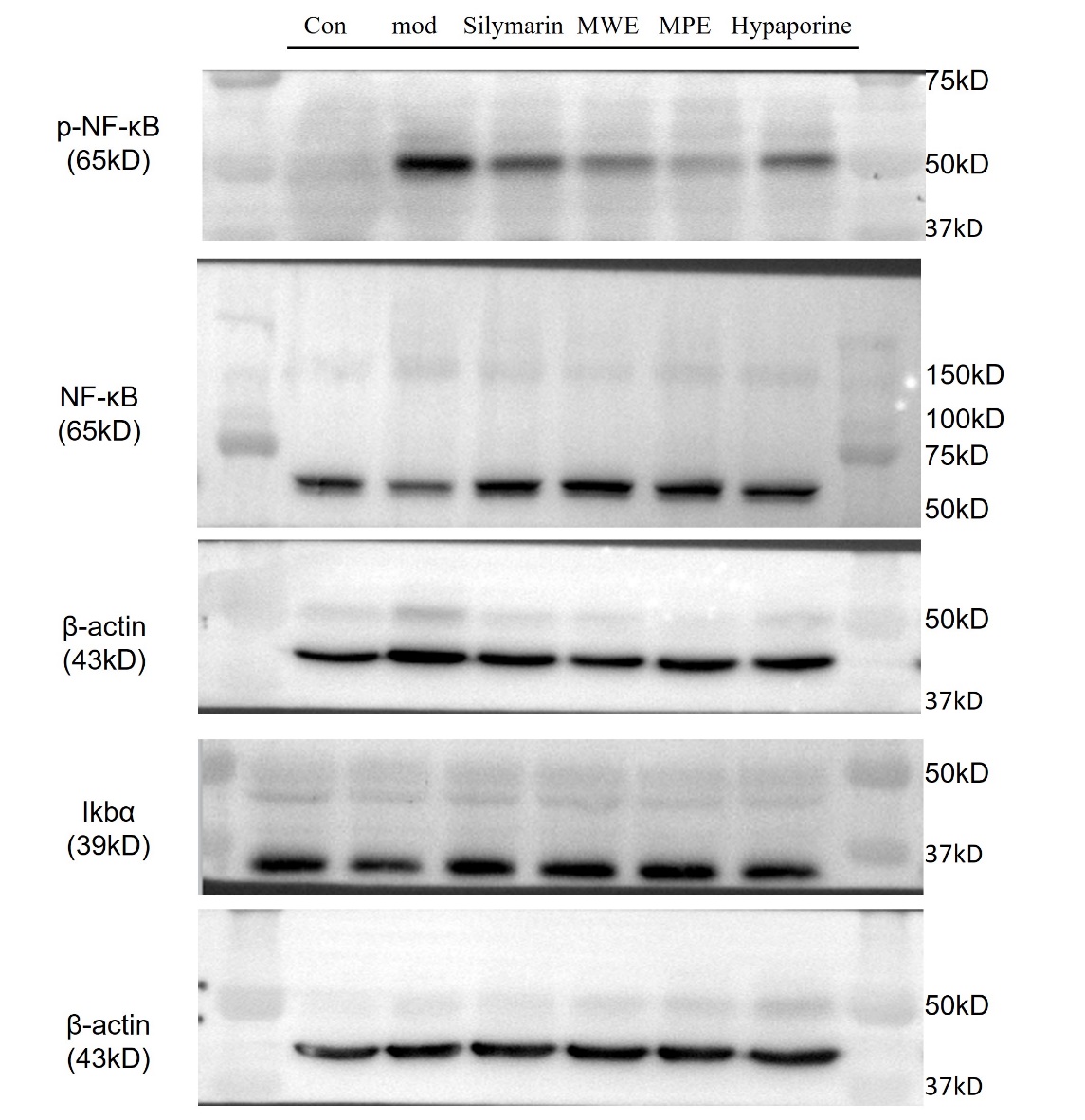


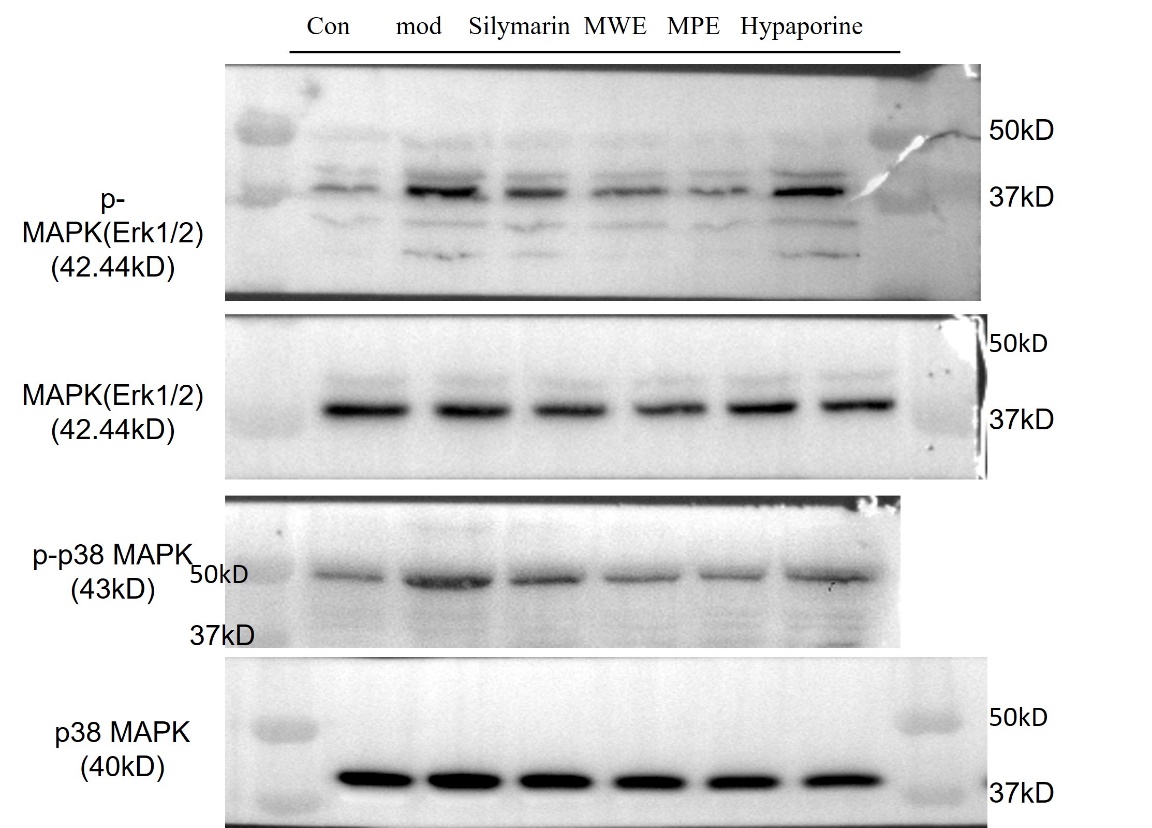


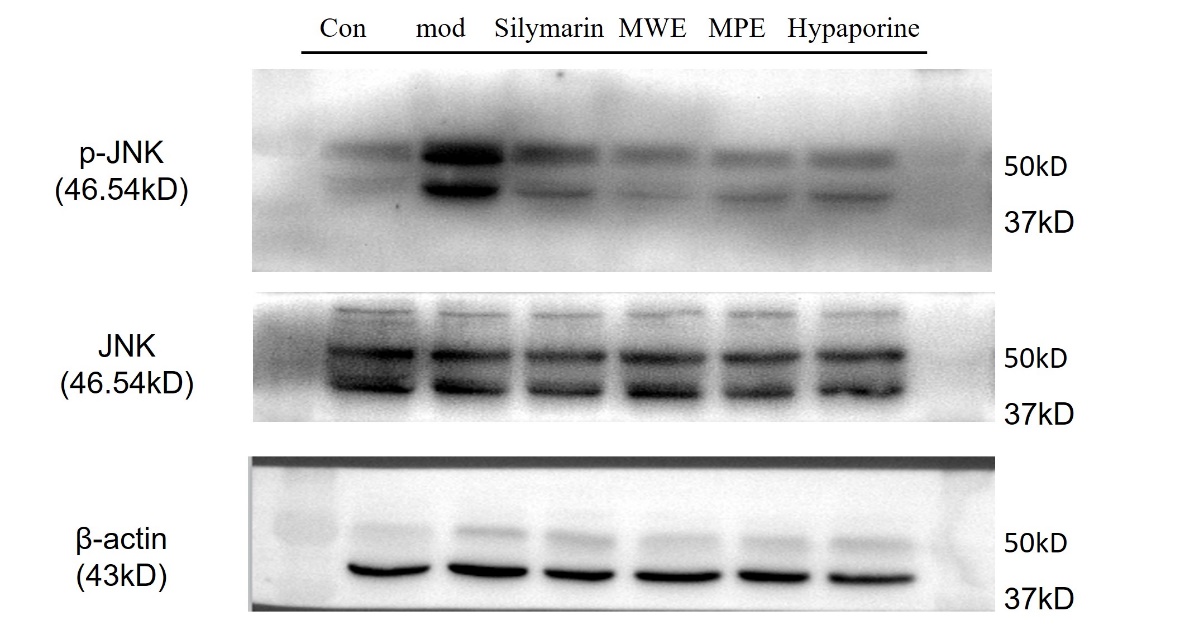


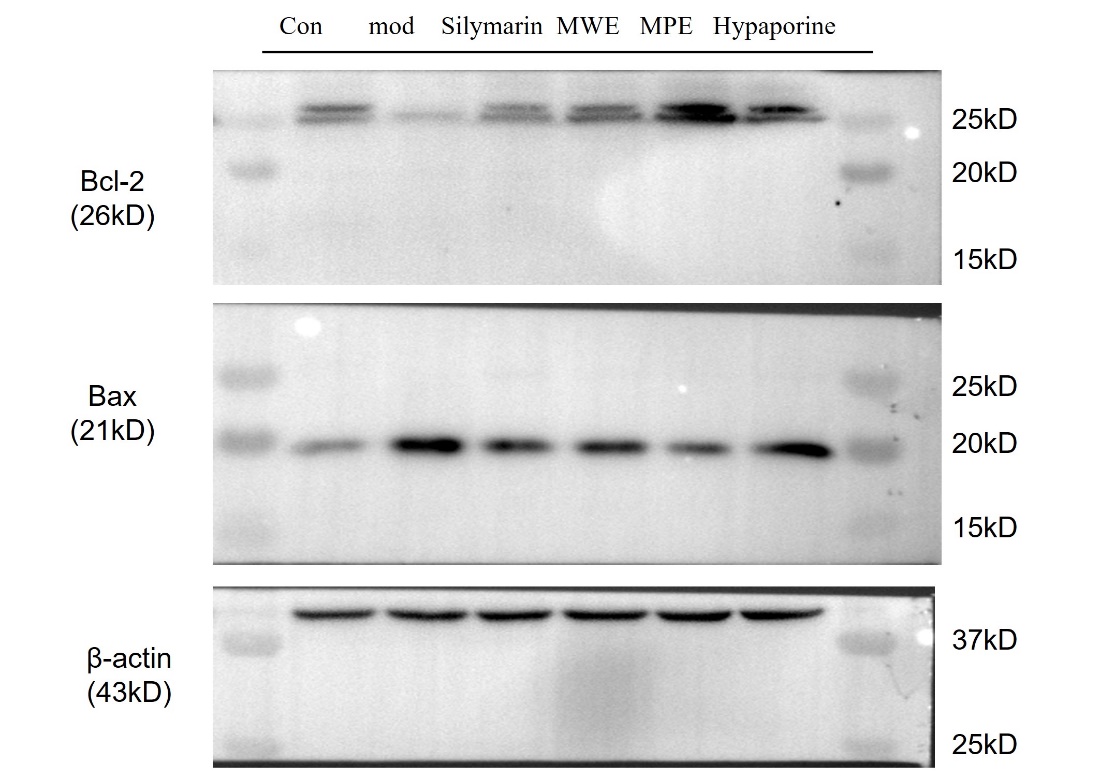

Supplement: Supplementary file 1 — Figure S1. Figure S2. [file FSN3-11-7026-s001.docx]
